# Influence of leptin and its receptors on individuals under chronic social stress behavior

**DOI:** 10.3389/fendo.2024.1281135

**Published:** 2024-02-01

**Authors:** Renata M. F. Mélo, Rafaela S. Barbosa, Victória L. Ozório, Gabriel M. Oliveira, Samuel I. M. Horita, Andrea Henriques-Pons, Tânia C. Araújo-Jorge, Viviane M. S. Fragoso

**Affiliations:** ^1^ Laboratory of Innovations in Therapies, Education and Bioproducts, Fundação Oswaldo Cruz, Rio de Janeiro, RJ, Brazil; ^2^ Laboratory of Cell Biology, Fundação Oswaldo Cruz, Rio de Janeiro, RJ, Brazil; ^3^ Laboratory on Thymus Research, Fundação Oswaldo Cruz, Rio de Janeiro, RJ, Brazil

**Keywords:** leptin, model of spontaneous aggression, chronic stress, central nervous system, leptin receptor (LEPR)

## Abstract

Stress is the body’s physiological reaction to a dangerous or threatening situation, leading to a state of alertness. This reaction is necessary for developing an effective adaptive response to stress and maintaining the body’s homeostasis. Chronic stress, caused mainly by social stress, is what primarily affects the world’s population. In the last decades, the emergence of psychological disorders in humans has become more frequent, and one of the symptoms that can be observed is aggressiveness. In the brain, stress can cause neuronal circuit alterations related to the action of hormones in the central nervous system. Leptin, for example, is a hormone capable of acting in brain regions and neuronal circuits important for behavioral and emotional regulation. This study investigated the correlation between chronic social stress, neuroendocrine disorders, and individual behavioral changes. Then, leptin and its receptors’ anatomical distribution were evaluated in the brains of mice subjected to a protocol of chronic social stress. The model of spontaneous aggression (MSA) is based on grouping young mice and posterior regrouping of the same animals as adults. According to the regrouping social stress, we categorized the mice into i) harmonic, ii) attacked, and iii) aggressive animals. For leptin hormone evaluation, we quantified plasma and brain concentrations by ELISA and evaluated its receptor and isoform expression by western blotting. Moreover, we verified whether stress or changes in leptin levels interfered with the animal’s body weight. Only attacked animals showed reduced plasma leptin concentration and weight gain, besides a higher expression of the high-molecular-weight leptin receptor in the amygdala and the low-molecular-weight receptor in the hippocampal region. Aggressive animals showed a reduction in the cerebral concentration of leptin in the hippocampus and a reduced high-and low-molecular-weight leptin receptor expression in the amygdala. The harmonic animals showed a reduction in the cerebral concentration of leptin in the pituitary and a reduced expression of the high-molecular-weight leptin receptor in the amygdala. We then suggest that leptin and its receptors’ expression in plasma and specific brain areas are involved in how individuals react in stressful situations, such as regrouping stress in MSA.

## 1 Introduction

According to the literature, stress is a physiological reaction of the body in the face of a situation of danger or threat caused by a psychological, environmental, or physiological stimulus. Then, the individual enters a state of alert, causing physical or emotional changes (1). This physiological reaction is necessary for developing an effective adaptive response to stress and, consequently, restoring homeostasis (2). The type of stress varies according to the duration and intensity of the exposure to the stressful event. Acute stress occurs when an individual is exposed to an intense stressor for a short duration. In contrast, chronic stress develops when the individual is exposed to a stressor with lower intensity for an extended period (3).

Chronic stress has become the most prevalent issue affecting the global population, particularly in densely populated urban centers, primarily stemming from social stressors. Social stress can manifest in various ways, such as social conflicts due to socioeconomic disparities, work-related stress, and, more recently, the prolonged social isolation induced by the COVID-19 pandemic (4–6). Individuals exposed to chronic social stress may experience significant physical and physiological repercussions, ultimately impacting brain functions and leading to changes in the neuroendocrine system (7). Exposure to chronic social stress can also lead to psychic damage, becoming a stimulus for the emergence of psychological disorders, such as anxiety, panic disorder, depression, and posttraumatic stress disorder (3, 6).

However, some individuals manage to adapt to stressful situations healthily, demonstrating resilience (8). According to the American Psychological Association, resilience is a recuperative process that involves difficult experiences and the ability to adapt to traumas, tragedies, threats, or significant sources of stress (9).

Unfortunately, the development of mental disorders in humans has become more frequent and one of the symptoms that may appear is aggressive behavior. Aggressiveness is a behavior in which an individual intends to cause physical or mental harm to another of its species (10). This behavior may appear in different forms, such as impulse aggression which has a strong emotional character but lower intensity, causing exacerbated aggression (11). Constant impulsive aggressive behavior can be considered pathological, leading the individual to commit acts of violence.

In mice used in scientific research, the study of violence is conducted by analyzing aggressive behavior (12). There is currently no consensus on the causes of aggression in laboratory animals, as it involves a complex network of factors related to genetics, biochemistry, neuroanatomy, environment, and physiology that determine the occurrence of escalated aggression episodes within a specific group of animals (13, 14).

The model of spontaneous aggression (MSA) is based on grouping young mice and regrouping in adulthood. Regrouping is considered a stressful stimulus for these animals, and some individuals react with aggressive behavior, while others respond with harmonic behavior (15, 16). Stress can cause alterations in neural circuits, brain structures, and hormone action or regulation in the central nervous system (CNS), including hormones such as leptin. Leptin is produced by adipocytes and released in the bloodstream (17, 18). In the CNS, leptin binds to its receptors in several cerebral regions and can regulate feeding and energetic homeostasis (19).

Increased fatty tissue in the organism stimulates the production and bloodstream release of leptin (19). This hormone acts mainly in the hypothalamus, stimulating anorexigenic hormones, such as pro-opiomelanocortin (POMC), and reducing food intake. In addition, leptin acts in energetic homeostasis by regulating lipid and carbohydrate metabolism in peripheral tissues (18).

Furthermore, leptin also acts in the ventral tegmental area (VTA), reducing dopaminergic neuronal firing from the VTA to mesolimbic dopaminergic areas (20). The mesolimbic dopaminergic pathway is responsible for the regulation of pleasure and reward sensations. A decrease in the dopamine release leads to a reduction in the stimulus to feed. However, the action of leptin in this pathway may influence emotional and behavioral regulation because the regions responsible for these regulations, such as the amygdala and hippocampus, are involved in the mesolimbic pathway (21).

In this work, we aimed to investigate the correlation among social chronic stress, neuroendocrine disorders, and behavioral changes using leptin and leptin receptor analysis in the brains of mice under social chronic stress.

## 2 Materials and methods

### 2.1 Animals

Male Swiss Webster mice (n= 30/assay) were obtained from the Multidisciplinary Center for Biological Research in the Area of Laboratory Animal Science (CEMIB/UNICAMP) and housed at our institutional animal facility. Animals adapted to the environment for seven days under stable temperature (20–22°C), humidity (40–60%), and noise (60 Db) conditions with twelve-hour light/dark cycles regulated by ventilated shelves. Food and water were offered *ad libitum*. All studies with animals were revised and approved by the Animal Use Ethics Committee of the Oswaldo Cruz Institute (CEUA/IOC) under license number CEUA/IOC-032/2019-A1.

### 2.2 Model of spontaneous aggression

**Figure 1:**
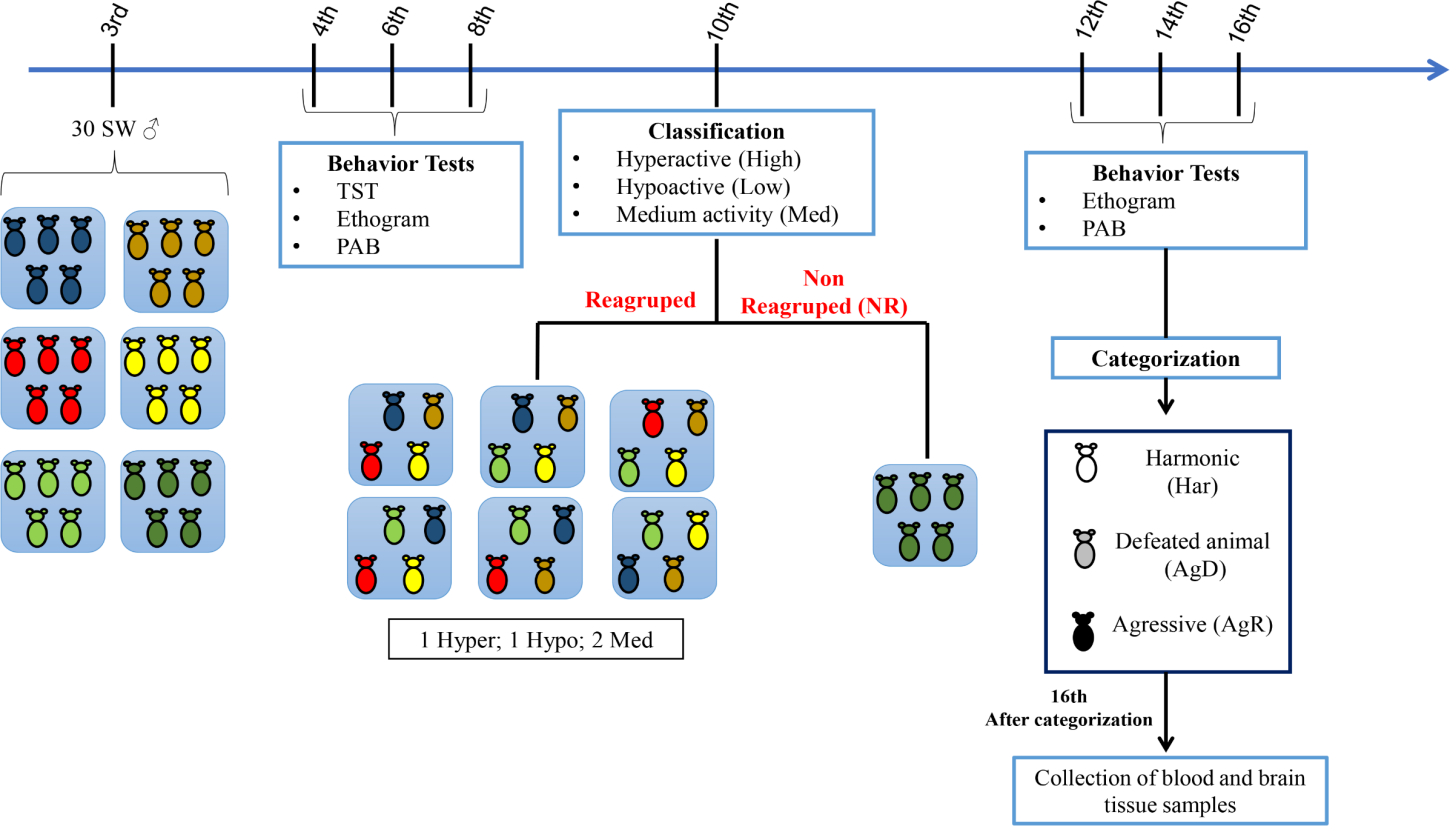
Experimental design of the model of spontaneous aggression (MSA) The male Swiss Webster mice were divided into five groups at the 3rd week of life (n=5 in each group), and behavioral tests, including the tail suspension test (TST), ethogram and pattern of aggressive behavior (PAB) test, were performed at the 4th, 6th and 8th weeks of life. At the 10th week of life, according to their mobility in the TST, the mice were classified as hyperactive (High), hypoactive (Low) or medium mobility (Med) and selected for regrouping. Most of the animals were regrouped into five groups with one High animal, one Low animal and three Med animals in each group. Regrouping represents a situation of social stress for animals. One group of 5 animals was not regrouped (NR), they were the social stress negative control. In the 12th, 14th and 16th weeks of life, we performed the aggressive ethogram and the PAB test. In the 16th week of life, the regrouped animals were categorized as harmonic (Har; social stress positive control), defeated (AgD) or highly aggressive (AgR). Then, the blood samples and cerebral regions were collected for ELISA and Western blot analysis.

At three weeks old (wko), mice were divided into six groups (A1 to A6), with five animals individually identified in each cage (C1 to C5). At the fourth, sixth, and eighth wko, we performed the tail suspension test (TST), described by Steru et al. (1985). This test was done to map the activity profile of each animal during the grouping; then, an ethogram was generated, and an aggressive behavior (PAB) test was also performed. At the tenth wko, the animals were regrouped according to the TST results into six groups (R1-R6), with one hyperactive individual (hyper), one hypoactive individual (hypo), and two individuals with median activity in each cage. One cage with five animals was not regrouped (NR), which became the negative control. At the twelfth, fourteenth, and sixteenth wko, the ethogram and PAB were repeated, and at the sixteenth wko, the animals were categorized according to their behavior. Harmonic (Har) animals were those with reduced aggression and discrete or absent body lesions; Har animals were considered resilient individuals and became our positive control. Attacked animals (AgD) were those showing moderate and intense body lesions caused by being subjected to aggression. Aggressive animals (AgR) were those with highly aggressive behavior against the other animals in the cage. After categorization, all animals were euthanized, and brain tissues were collected (**Figure 1**).

### 2.3 Tail suspension test

The final third of their tail suspended the animals and the time for which animals stayed paralyzed during the five-minute test was computed. Three classifications were defined according to the physical reaction of each animal: a) low activity (hypoactive), 104 to 150 seconds of immobility; b) median active, 51 to 103 seconds of immobility; and c) high active (hyperactive), 0 to 50 seconds of immobility (22).

### 2.4 Ethogram and pattern of aggressive behavior

The ethogram and PAB tests were performed in all animals at grouping and regrouping. The ethogram was based on recordings of each cage (top view) using a Canon PowerShot SX20 IS^®^ (Lake Success, New York, USA) camera for 30 minutes. An ethogram allowed for identifying aggressive, attacked, and harmonic animals; quantifying numbers of attacks; and measuring the extent of lesions (cm²) with a digital caliper (Digimess-, China). The PAB test was performed through an individual physical inspection of all animals. The physical inspection was quantified according to predetermined scores: 0, no body lesions; 1, soft nonsexual lesions on the back or tail; 2, moderate lesions on the tail, back, or scrotum caused by aggressive events; and 3, severe lesions on the back, tail, and scrotum caused by aggressive events.

### 2.5 Body weight

All animals were weighed during the grouping phase (fourth, sixth, and eighth wko) and the regrouping phase (twelfth, fourteenth, and sixteenth wko) using a precision analytical balance Bel S2202H (São Paulo-BR). The data were analyzed in spreadsheets using the Excel application (Microsoft 365, version 18.2210.1203.0), where we quantified the weight variation by calculating the difference between the average weight of each animal before and after regrouping.

### 2.6 ELISA

To analyze plasma leptin levels, all animals were anesthetized with xylazine (10 mg/kg) and ketamine (75 mg/kg). Then, blood samples were collected by cardiac puncture. The samples were immediately centrifuged for 10 min at 1000 x g and 4°C for cell remotion and centrifuged again for fifteen minutes at 2000 x g, and the supernatant was collected. For cerebral leptin analysis, each animal’s pituitary gland, hippocampus, amygdala, and prefrontal cortex regions were collected, sonicated in lysis buffer (5 M guanidine-HCl diluted in 50 Mm Tris, Ph 8.0 + 1X PBS supplemented with 1X protease inhibitor cocktail), and processed according to the manufacturer’s instructions. We used a Leptin Mouse ELISA Kit (KMC2281, Thermo Fisher Scientific, Massachusetts, USA) to analyze cerebral tissue and plasma concentrations of the hormone. The results were obtained by a Glomax Multi Spectrophotometer (Promega) at 450 nm. The relative leptin levels in the brain regions were normalized to the relative expression of leptin in the same brain regions in the NR animals.

### 2.7 Western blotting

As described by (23), the NR, Har, AgD and AgR animals were anesthetized with xylazine (10 mg/kg) and ketamine (75 mg/kg). Then, the euthanasia was induced by overdose with intraperitoneally injected xylazine (10 mg/kg) and ketamine (100 mg/kg) in 0.01 Ml (needle: 20x5.5 mm). After euthanasia, the brain tissue was dissected, and the hippocampus, hypothalamus, amygdala, and prefrontal cortex were collected. The samples were homogenized with lysis buffer for protein extraction supplemented with protease and phosphatase inhibitors (Sigma‒Aldrich, San Luis, USA). The samples (n=10 per category) were centrifuged (five minutes, 96 xg, room temperature), and protein levels were determined through spectrophotometry using a bicinchoninic acid (BCA) kit (Invitrogen, Massachusetts, USA). Subsequently, the samples were diluted in sample buffer (80 Mm Tris-HCl Ph 6.8, 2% SDS, 12% glycerol, 5% β-mercaptoethanol, and 0.05% bromophenol blue) at 100°C for five minutes and placed on cooled plates. The proteins were separated on a 10% polyacrylamide gel with sodium dodecyl sulfate (SDS‒PAGE) containing 20 µg of protein in each well and transferred to a polyvinylidene difluoride (PVDF) membrane.

The membranes were incubated for one hour at room temperature with a blocking solution (5% skimmed milk in 0.1% TBT-T). Then, the membranes were incubated with anti-GAPDH (protein load control) (Fitzgerald, code 10R-G109A) 1:70,000 at room temperature for thirty min or with a primary anti-leptin receptor (LepR) antibody made in goat (0.1:1,000) (Invitrogen, Código PA5-18522) overnight at 4°C, both diluted in blocking solution. The membranes were incubated with HRP-conjugated secondary anti-mouse or anti-goat antibodies for thirty min and one hour, respectively, at room temperature and both diluted 1:10,000 in blocking solution. The membranes were washed three times with washing buffer (0.1% TBS-T) between each step. Peroxidase was developed for chemiluminescence using the Super Signal West Pico kit (Invitrogen). Densitometry was performed using Evolution-CAPT for Fusion FX6 Edge and Solo 6s Edge software. The relative expression of the leptin receptors was normalized using GAPDH. All values were expressed as variation index (VI).

### 2.8 Statistical analysis

Data are presented as the mean ± SD or SE values for each condition, as depicted in figure legends. D’Agostino-Pearson normality tests were performed for all values to assess their Gaussian distribution. Comparisons between groups were performed by nonparametric Kruskal‒Wallis tests followed by Dunn’s multiple comparisons tests using GraphPad Prism software version 8.0.1 for Windows (GraphPad Software, USA). Differences of p ≤ 0.1 were considered significant. The analysis was performed in a double-blind manner.

## 3 Results

### 3.1 Categorization of aggressive animals

**Figure 2:**
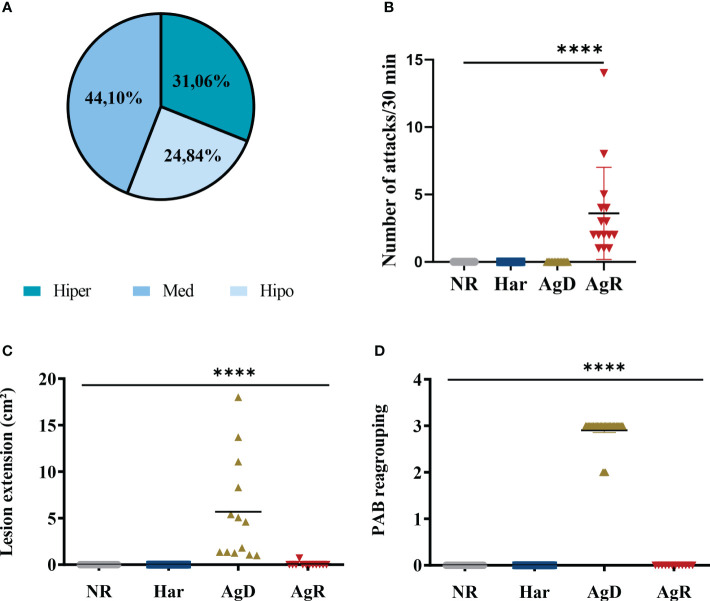
Evaluation of behavioral parameters in social stress. **(A)** Distribution of animals according to the mobility profile obtained by the TST for regrouping in the 10th week of life. The groups were hyperactive (31,06%, dark blue), medium (44,10%, blue), and hypoactive (24,84%, light blue). **(B)** Number of attacks/30 min after regrouping, according to the categorization of animals into not regrouped (NR, gray circle), harmonic (Har, dark blue square), defeated (AgD, green triangle) and aggressive (AgR, inverted red triangle) groups. **(C)** Lesion extent (cm2) after regrouping according to the behavioral categories of NR, Har, AgD and AgR animals. **(D)** Evaluation of the pattern of aggressive behavior (PAB) after regrouping according to the behavioral categories of NR, Har, AgD and AgR animals. The PAB test was performed through an individual physical inspection of all animals, enabling the quantification of aggression according to predetermined scores: 0, no bites or injuries on the body; 1, reduced aggressive nonsexual events and discrete bite signs on the back or tail; 2, aggressive events and moderate lesions on the tail, back and scrotum; and 3, severe events of aggression and marked lesions on the tail, back and scrotum. The values are expressed according to the individual results of the mice from three independent experiments (NR, n= 15; Har, n= 20; AgD, n= 15; AgR, n=15 animals). **** corresponds p < 0.0001 between AgR and the other categories and AgD and the other categories by Kruskal-Wallis and Dunn's tests.

The selection of highly aggressive animals was conducted using the MSA, as depicted in **Figure 1**. The TST, ethogram, and PAB were also performed during the grouping step at four, six, and eight wko. The mobility analysis was obtained by the TST, with 31.06% of hyperactive animals (fourth wko: 11.68 seconds (s)± 10.67 s; sixth wko: 21.32 s ± 19.23 s; eighth wko: 38.20 s ± 30.26 s), 44.10% with median activity (fourth wko: 41.37 s ± 30.36 s; sixth wko: 87.68 s ± 31.30 s; eighth wko: 94.64 s ± 37.31 s), and 24.84% hypoactive animals (fourth wko: 84.47 s ± 39.6 s; sixth wko: 140.92 s ± 34.36 s; eighth wko: 146.18 s ± 36.86 s) (**Figure 2A**). Neither aggressive behavior nor body lesions were observed. After regrouping, the ethogram and PAB tests were conducted again, and the animals were divided according to their aggressiveness: harmonic (Har), attacked (AgD), or aggressive (AgR) animals. After repeating the ethogram, we noticed a significant increase in the number of attacks by AgR animals with 3.6 attacks/30 minutes 0.9 (p <0.0001) compared to other categories (NR: 0.0: Har: 0.0 ± 0.0; AgD: 0.0 ± 0.0 attacks/30 min) (**Figure 2B**). In addition, the AgD animals showed a significant increase in lesion size of 5.69 ± 1.54 cm² (p <0.0001) (**Figure 2C**) and an increase in the PAB test score of 2.9 ± 0.05 cm² (p <0.0001) (**Figure 2D**) compared to animals in the other categories.

### 3.2 Body weight reduction after social stress

**Figure 3:**
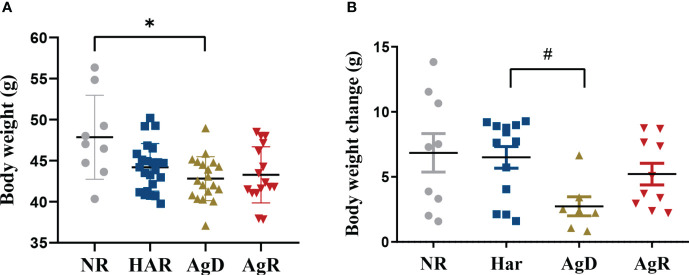
Evaluation of animal body weight under social stress. **(A)** Body weight of animals after being subjected to the social stress of regrouping according to the categorization of animals into not regrouped (NR, gray circle), harmonic (Har, dark blue square), defeated (AgD, green triangle) and aggressive (AgR, inverted red triangle) groups. **(B)** Variation in body weight of NR, Har, AgD, and AgR animals, according to the body weight change of each animal before (4th, 6th, and 8th weeks of age) and after (12th, 14th and 16th weeks of age) regrouping. The values are expressed according to the individual results of the mice from two independent experiments (NR, n= 10; Har, n= 12; AgD, n= 19; AgR, n=16 animals). * corresponds to p < 0.05 between AgD and NR, and #corresponds to p < 0.1 between AgD and Har by Kruskal-Wallis and Dunn's tests.

To evaluate whether the stress caused by MSA regrouping influenced body weight, the animals’ weight was recorded throughout the MSA experiment at the 4^th^, 6^th^, 8^th^, 12^th^, 14^th^, and 16^th^ weeks of life. We observed a significant decrease of 42.81 ± 0.6 g (*p* ≤ 0.05) in the body weight of attacked animals compared with the negative control (NR: 47.85 ± 1.7; Har: 44.20 ± 0.6; AgR: 43.28 ± 0.8 g) (**Figure 3A**). Additionally, the number of animals exhibiting a change in body weight after social stress was also analyzed. For this, the mean weight of each individual in the grouping phase (fourth, sixth, and eighth wko) was compared with the mean weight after regrouping (twelfth, fourteenth, and sixteenth wko). Animals categorized as AgD also showed a lower weight variation of 2.41 ± 0.7 g (p ≤ 0.1) compared to harmonic animals (NR: 6.85 ± 1.5; Har: 6.04 ± 0.9; AgR: 5.21 ± 0.84 g) (**Figure 3B**).

### 3.3 Chronic social stress interferes with plasma and cerebral leptin concentrations

**Figure 4:**
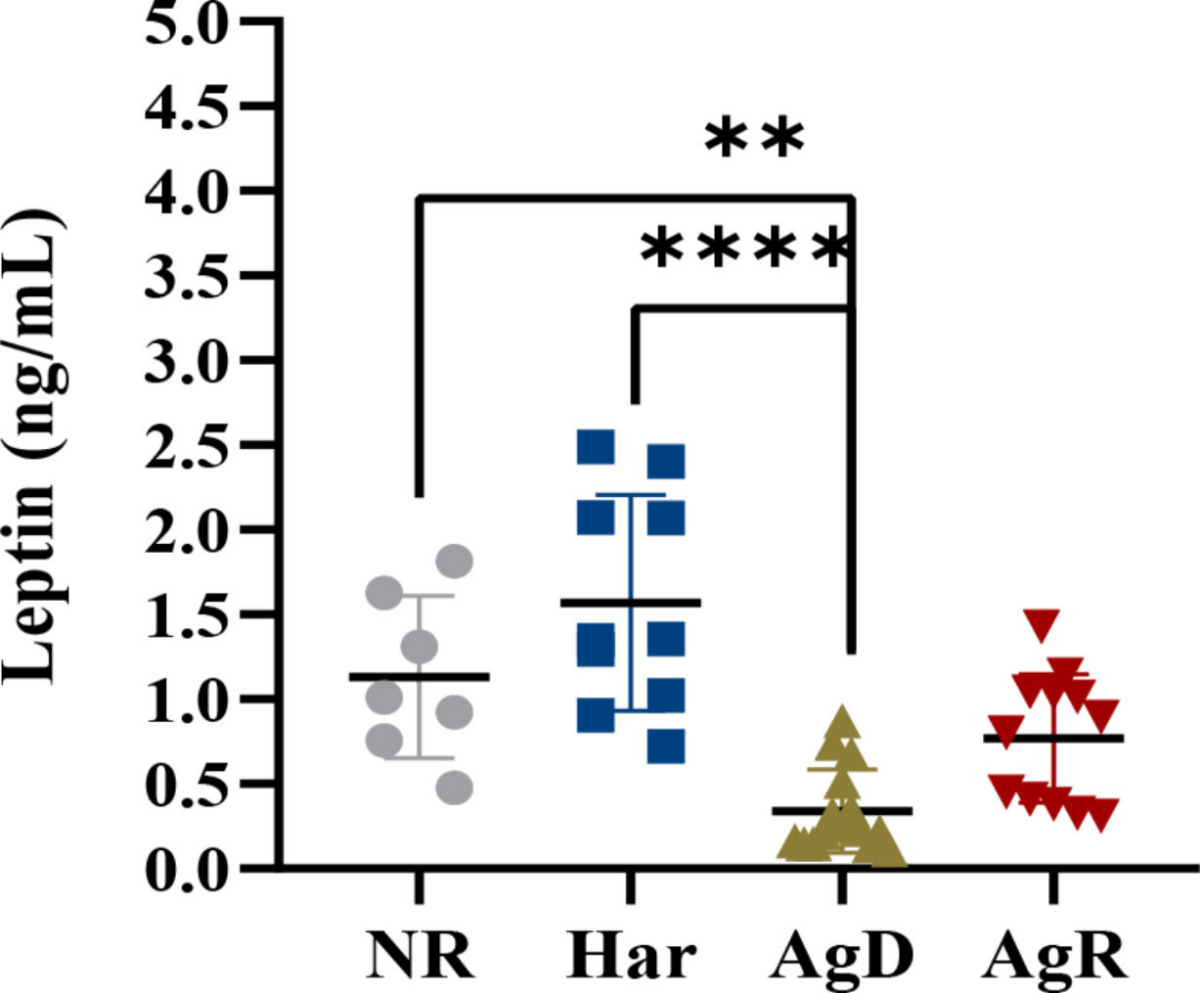
Evaluation of plasma leptin concentrations after social stress. Plasma leptin concentrations in not regrouped (NR, gray circle), harmonic (Har, dark blue square), defeated (AgD, green triangle) and aggressive (AgR, inverted red triangle) animals subjected to social regrouping stress. The values are expressed according to the individual results of the mice from two independent experiments (NR, n= 10; Har, n= 10; AgD, n= 13; AgR, n=14 animals). ** corresponds to p < 0.01 between AgD and NR. **** corresponds to p < 0.0001 between AgD and Har by Kruskal-Wallis and Dunn's tests.

**Figure 5:**
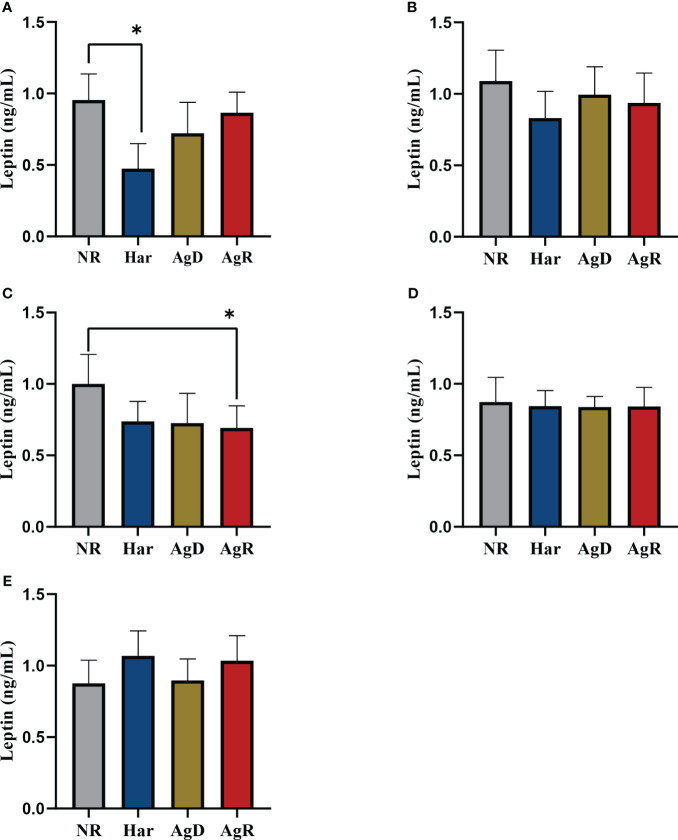
Evaluation of the levels of cerebral leptin. Quantitative analysis of cerebral leptin expression by ELISA according to the categories NR (gray bar), Har (blue bar), AgD (green bar) and AgR (red bar) in the **(A)** pituitary gland, **(B)** hypothalamus, **(C)** hippocampus, **(D)** amygdala, and **(E)** prefrontal cortex. The values are expressed according to the individual results of the mice from two independent experiments (NR, n= 10; Har, n= 10; AgD, n= 13; AgR, n=14 animals). * corresponds to p < 0.05 between Har and NR, and AgR and NR by Kruskal-Wallis and Dunn's tests.

Exposure to social stress can cause changes in the concentration and production of hormones, such as leptin (18). We investigated whether social stress induced by MSA can promote changes in plasma leptin concentration in animals subjected to MSA. ELISA analysis showed a significant decrease in plasma leptin concentration in AgD animals (0.339 ± 0.245 ng/ml) compared with positive (p ≤0.0001) and negative (p ≤0.01) controls (NR: 1.131 ± 0.478; Har: 1.568 ± 0.637; AgR: 0.770 ± 0.397 pg/Ml) (**Figure 4**) (**Supplementary 1**). This hormone primarily acts in the brain, helping regulate food intake (20). Thus, we also investigated by ELISA whether animals submitted to MSA have alterations of leptin levels in brain regions, including the pituitary gland, hypothalamus, hippocampus, amygdala, and prefrontal cortex. Interestingly, Har animals presented a significant decrease in leptin concentration in the pituitary gland 0.42 ± 0.17 (p ≤0.05) ng/ml (pituitary gland: NR: 0.95 ± 0.18; AgD: 0.72 ± 0.22; AgR: 0.86 ± 0.14 ng/ml) and only AgR animals presented a significant decrease in leptin concentration in the hippocampus with and 0.69 ± 0.16 ng/ml (Hippocampus: NR: 1.00 ± 0.21; Har: 0.74 ± 0.14; AgD: 0.73 ± 0.21 ng/ml) (**Figures 5A–E**), both compared with the NR. (**Figures 5**) (**Supplementary 2**).

### 3.4 Chronic social stress induced by MSA influences leptin receptor expression in brain regions

**Figure 6:**
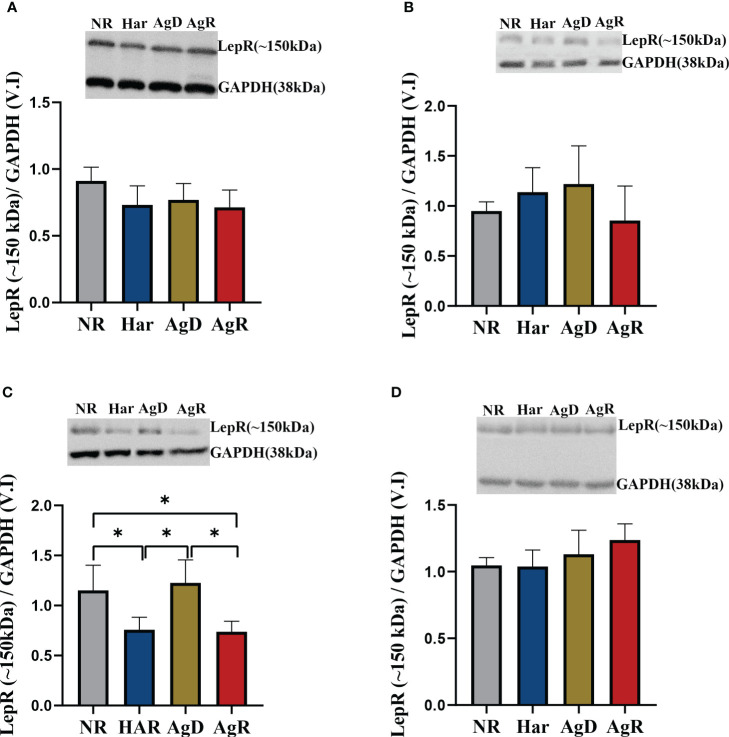
Expression levels of the high-molecular-weight leptin receptor. Quantitative analysis of high-molecular-weight leptin receptor expression by Western blotting analysis according to the categories NR (gray bar), Har (blue bar), AgD (green bar) and AgR (red bar) in the **(A)** hypothalamus, **(B)** hippocampus, **(C)** amygdala, and **(D)** prefrontal cortex. GAPDH was used as an internal control for the samples. The values are expressed as the variation index (V.I.) of the means ± standard deviations (SDs) of the analyzed samples from three independent experiments using the Kruskal-Wallis and Dunn's test (NR, n=9; Har, n=10; AgD, n=15; AgR, n=10 animals). * corresponds to statistical significance (p < 0.05).

The leptin hormone binds to its receptor in the brain to perform its functions. Thus, we investigated how chronic social stress can affect leptin receptor expression in the hypothalamus, hippocampus, amygdala, and prefrontal cortex. There are six isoforms of leptin receptors, denoted as a, b, c, d, e, and f. Among these, only isoform b has a high molecular weight and can effectively transmit the signal of leptin binding to the cell (17). In this study, due to the antibody used, we were able to observe only two bands in the western blot, one well-formed at ~100 kDa, and another also well-formed at ~150 kDa. As a result, we divided the isoforms of LepR into low molecular weight LepR (~100 kDa) and high molecular weight LepR (~150 kDa). Only the amygdala showed alterations in high-molecular-weight leptin receptor expression; we observed a significant decrease of 0.74 ± 0.05 in AgR animals compared with AgD and NR animals (**Figure 6C**). Har animals also showed a decrease in high-molecular-weight receptors of 0.76 ± 0.05 compared with the negative control (NR: 1.15 ± 0.10). However, the AgD animals showed an increase in this receptor of 1.23 ± 0.10 (p ≤0.05) compared with Har and AgR animals (**Figure 6**) (**Supplementary 2**).

**Figure 7:**
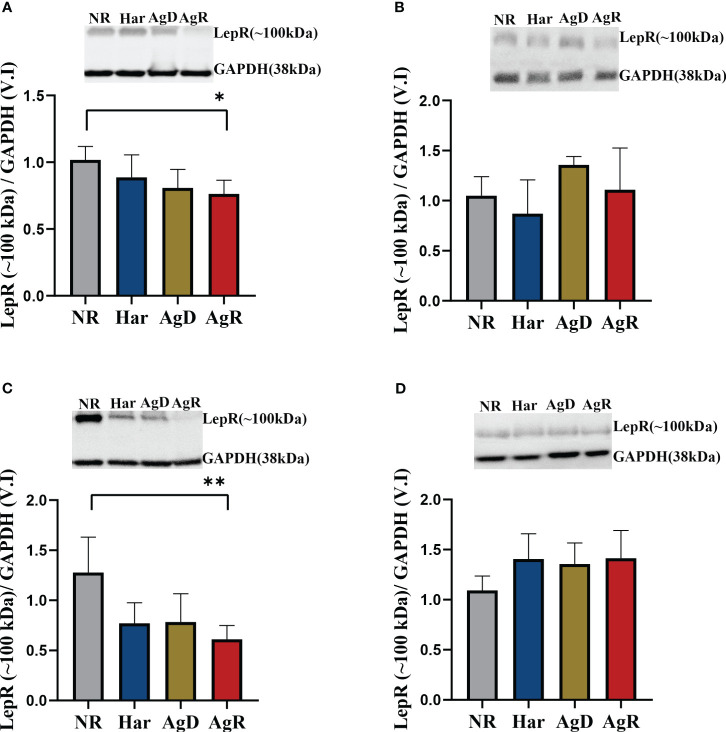
Expression levels of the low-molecular-weight leptin receptor. Quantitative analysis of low-molecular-weight (~100 kDa) leptin receptor expression by Western blotting analysis according to the categories NR (gray bar), Har (blue bar), AgD (green bar) and AgR (red bar) in the **(A)** hypothalamus, **(B)** hippocampus, **(C)** amygdala, and **(D)** prefrontal cortex. GAPDH was used as an internal control for the samples. The values are expressed as the variation index (V.I.) of the means ± standard deviations (SDs) of the analyzed samples from three independent experiments using the Kruskal-Wallis and Dunn's test (NR, n=9; Har, n=10; AgD, n=15; AgR, n=10 animals). * corresponds to p < 0.05 between AgR and NR; ** corresponds to p < 0.01 between AgR and NR.

When we analyzed low-molecular-weight leptin receptor expression, alterations in the amygdala and hypothalamus were observed (**Figures 7B, C**). Only AgR animals showed a decrease in low-molecular-weight receptor expression of 0.61 ± 0.06 and 0.76 ± 0.04 (p ≤0.05), respectively, compared with the NR group (Amygdala: NR: 1.28 ± 0.14; Har: 0.77 ± 0.08; AgD: 0.78 ± 0.11. Hypothalamus: NR: 1.02 ± 0.04; Har: 0.88 ± 0.06; AgD: 0.81 ± 0.05) (**Figure 7**) (**Supplementary 2**).

**Figure 8:**
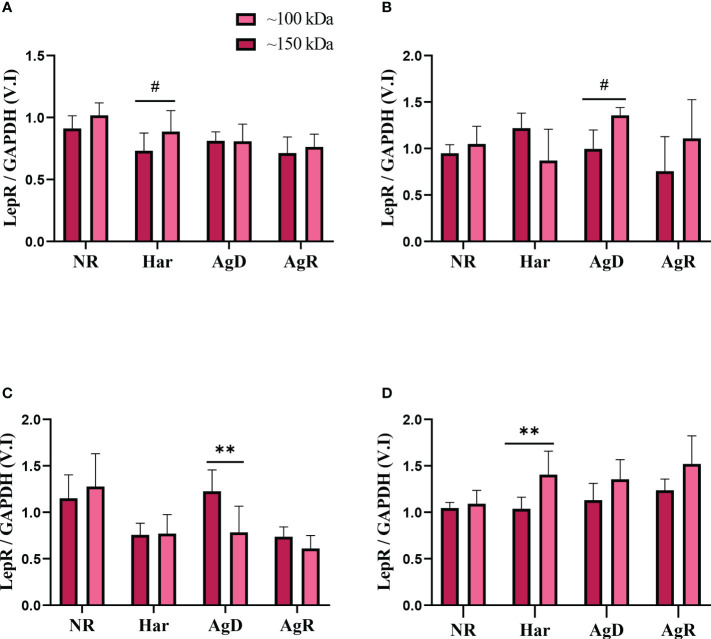
Analysis of leptin receptor expression levels according to molecular weight. Comparing leptin receptor expression levels of high (dark pink bars) and low molecular weight (light pink bars), according to the categories of NR, Har, AgD, and AgR animals, in the **(A)** hypothalamus, **(B)** hippocampus, **(C)** amygdala, and **(D)** prefrontal cortex. The values are expressed according to the indivi- dual results of the mice from three independent experiments. ** corresponds to p <0.01, and # corresponds to p <0.1 by Kruskal-Wallis and Dunn's tests.

Thus, we decided to compare the expression of these two leptin receptors between the animal groups in each cerebral region investigated. Curiously, only the AgD and Har groups had alterations between these two leptin receptor types. A significant increase in low-molecular-weight leptin receptors was observed in Har animals in the prefrontal cortex and hypothalamus (Prefrontal cortex: low m. weight: 1.41 ± 0.2; high m. weight: 1.04 ± 0.1; p ≤0.05. Hypothalamus: low m. weight: 0.89 ± 0.17; high m. weight: 0.73 ± 0.14; p ≤0.1) (**Figures 8 A, B**). The AgD animals showed an increase in high-molecular-weight leptin expression in the amygdala (high m. weight: 1.23 ± 0.23; low m. weight: 0.78 ± 0.28; p ≤ 0.01) (**Figure 8C**). Moreover, an increase in low-molecular-weight leptin receptor expression was observed in AgD animals in the Hippocampus (low m. weight: 1.36 ± 0.08; high m. weight: 0.99 ± 0.20; p ≤ 0.1) (**Figure 8D**).

## 4 Discussion

Our research investigated whether chronic social stress can lead to changes in animal behavior by altering the levels of leptin hormone and its receptor in certain brain regions.

Stress is defined as the cumulative impact of external factors that lead to transient effects or disrupt the homeostasis (1). These transient effects can result in cerebral function alterations, stimulating the development of a balanced response to stressor stimuli (24, 25). The primary adaptive response of the CNS involves HPA axis activation, resulting in cortisol production and action. Stress stimulates the hypothalamus to produce and release corticotropin-releasing factor (CRF). CRF release stimulates the anterior pituitary gland to produce and secrete adrenocorticotrophic hormone (ACTH). ACTH released in the bloodstream stimulates the suprarenal gland to produce cortisol glucocorticoid (corticosterone in animals). Cortisol acts by blocking CRF and ACTH production and secretion by the hypothalamus and pituitary gland, respectively. Furthermore, cortisol has functional, structural, and molecular effects on the brain, leading to the remodeling of the neuronal architecture and an enhanced adaptation of the body to stress (26, 27).

However, there are brain regions capable of regulating the HPA axis, such as the amygdala and the hippocampus. These regions modulate the axis in different ways. The amygdala establishes neural projections to the hypothalamus, stimulating CRF production and secretion and initiating the HPA axis. However, the hippocampus also establishes neural projections to the hypothalamus, blocking CRF production and secretion and causing a reduction in HPA axis activity. In addition to these regions, hormones such as leptin can regulate the axis (20, 28).

Leptin is a hormone produced by adipose tissue and acts in the central nervous system. The hormone binds with its receptor and regulates food intake and energetic balance by inducing production of anorexigenic factors. By binding with its receptor, leptin can also regulate HPA axis activation by CRF and reduction of ACTH production and release in the hypothalamus and pituitary gland, respectively (17). Leptin has the ability to affect basal metabolism, reproductive function, and insulin secretion in the peripheral region (18).

Oliveira et al. (2014) observed that harmonic animals showed an increase in corticosterone levels. These animals were considered resilient and had a good adaptative stress response through HPA axis activation (29). We observed in this study that harmonic animals had reduced cerebral leptin concentration in the pituitary gland and hippocampus. The leptin reduction in these regions avoids causing an inhibitory effect on the HPA axis, leading to good axis function and enhanced body adaptation to stress.

We observed decreased plasma leptin concentration, body weight, and weight variation in attacked animals (AgD). However, no difference in cerebral leptin concentration was observed. As previously mentioned, leptin is synthesized by adipose tissue, and its primary function is to reduce food intake (18). Since leptin acts in the CNS by reducing food intake, leading to a decrease in adipose tissue, which reduces leptin production and secretion in the bloodstream. Resulting in a decrease in body weight and body weight variation in AgD animals. These animals are also constantly attacked, resulting in body lesions. In this case, the lesions would make it difficult for the locomotion to access the food, influencing weight gain and plasma leptin concentration observed in AgD animals.

Other studies show that alterations in plasma leptin concentration depend on the type of stress the individual was exposed to. In the case of chronic stress, such as the Social Defeat Test, exhibited reduced plasma leptin concentration (18, 30). This reduction can stimulate orexigenic hormone production to maintain the high energy demand for thermogenesis and other physiological functions. We believe that decreased plasma leptin levels in AgD animals can also regulate the energetic balance for thermogenic maintenance.

All organisms have leptin receptors. The leptin receptor has six different isoforms with different molecular weights. The LepRa (~101.058 kDa), LepRc (~100.789 kDa), LepRd (~101.863 kDa), and LepRf isoforms are crucial to ensure the passage of the hormone through the blood-brain barrier. The LepRe (~90 kDa) isoform is soluble and ensures leptin transportation from the bloodstream to target organs. The LepRb (~150 kDa) isoform is the only long isoform of this receptor, with an intracellular domain able to transmit the leptin-binding signal to the cell and produce an intracellular postsynaptic response (17, 18). In this work, the harmonic animals showed a reduction of high-molecular-weight leptin receptor expression in the amygdala. The amygdala can activate the HPA axis (20); thus, binding leptin to its receptors can block neural projections to the hypothalamus, inhibiting HPA axis activation. We suggest that the reduction of high-molecular-weight LepR in the amygdala can be a mechanism to avoid the inhibition of the axis in resilient animals.

In contrast, AgD animals showed increased expression of high-molecular-weight LepR in this same brain region. The amygdala is part of the temporal lobe of vertebrate brains. This cerebral region then belongs to the limbic system and it is considered an important area of aggressive regulation and sexual behavior, the formation of affective memories, identification of a dangerous situation, the perception and production of emotions, and the feeling of fear and anxiety. In this way, it triggers the individual to a state of alertness or anxiety when a stressor is perceived. In addition, it also acts as a regulator of body weight and food intake (31, 32). Thus, the inhibitory action of leptin in this region in AgD animals may be helping with its anorectic effect and regulation of emotional well-being.

A study found that intra-amygdalar injection of a low dose of leptin in male rats that showed anxious behavior was able to reverse this behavior through an increase in serotonin and glutamate, showing that leptin has anxiolytic properties (33). We believe that this anxiolytic action prevented the animals of the AgD group from noticing the attack promoted by the aggressor in advance. Therefore, making AgD animals more vulnerable to the attack, since the amygdala is responsible for identifying and signaling a dangerous situation.

Interestingly, the highly aggressive animals (AgR) showed a significant reduction in the concentration of leptin in the hippocampus, but without any alteration in the expression of LepR in this area. This result suggests that the difficulty in increasing corticosterone in AgR animals is not related to a possible inhibition of leptin in the hippocampus. Since there is a lower concentration of this hormone in the hippocampus, this area is free to carry out neuronal projections to the hypothalamus and perform negative feedback on the HPA axis. Besides that, the AgR animals showed a significant reduction in both high-molecular-weight and low-molecular-weight leptin receptors in the amygdala but without alteration in the cerebral concentration of leptin in any brain region. Leptin is capable of binding to other nonspecific receptors, such as the type 2 inhibitory dopaminergic receptor (D2R).

A study carried out using male mice showed that the administration of leptin and a D2R inhibitor led to depressive behavior in these animals, indicating that leptin can bind to this receptor to exert its antidepressant action (34). It is possible that in AgR animals, leptin may bind to D2R, since these animals showed reduced expression of LepR, thus avoiding the accumulation of this hormone in the amygdala. However, this connection may reduce neuronal activity in this region, thus reducing the feeling of satiety, pleasure, and reward and the ability to regulate aggressive behavior promoted by the amygdala. Consequently, the AgR animal would develop a compulsive aggressive behavior, seeking the feeling of satiety, pleasure, and reward.

We also observed a higher expression of the low-molecular-weight leptin receptor in the hippocampal region in AgD animals. The hippocampus is the region responsible for spatial orientation, especially memory. It is the region responsible for transforming short-term memories into long-term memories. In addition to inhibiting CRF secretion in the hypothalamus, it causes negative feedback on the HPA axis. Some studies show that leptin exhibits antidepressant action in the hippocampus through binding to the LepRb receptor. Guo and his team (2013) showed that LepRb isoform-deficient animals exhibited depressive behavior (35). Therefore, alterations in the expression of this isoform of the leptin receptor in the hippocampal region can cause more depressive behavior in the individual. We believe that AgD animals may tend to depressive behavior since these animals have more low-molecular-weight receptor isoforms than high-molecular-weight isoforms in the hippocampus, which may directly influence the lack of ability to react to the attacks suffered. This higher expression of low-molecular-weight isoforms may result from the chronic stress to which it is subjected or perhaps a genetic cause.

## 5 Conclusion

Our results suggest that changes in plasma and brain levels of the hormone leptin and its receptor isoforms influence the ability to develop a good adaptive response to stress and determine which behavior the individual will develop under a chronic social stress situation. Crucial alterations were observed in aggressive and attacked animals. Leptin receptor alterations were identified in the cerebral region of the amygdala, one of the regions involved in regulating the pleasure and reward sensations; identification of a dangerous situation; and emotional control, mainly of anxiety and fear. Furthermore, the expression of the high-molecular-weight isoform (LepRb), the only isoform capable of transmitting the leptin binding signal to the postsynaptic intracellular environment, showed the greatest change. Therefore, we observed that regrouping is a stress inducer in adult male mice, serving as a trigger for the development of behavioral changes, where some showed highly aggressive behavior, and others became resilient. The Har animals developed a good adaptive response to stress, as they had lower levels of leptin in region that is important for regulation of the HPA axis, such as the pituitary gland, and reduced LepRb in the amygdala. In this way, inhibition of the HPA axis was avoided, allowing for good production and secretion of corticosterone and the development of resilient behavior. On the other hand, the AgR animals showed alterations in the amygdala, where we observed a reduction in all LepR isoforms but without alteration in the cerebral leptin concentration. We suggest that the hormone binds to dopaminergic receptor 2, an inhibitory receptor. This may cause a reduction in the activation and functionality of this region, causing a decrease in the sensations of pleasure and reward and regulation of aggressive behavior. This results in the development of compulsive aggressive behavior. Interestingly, the AgD animals showed a reduction in plasma leptin concentration, weight, and weight variation, with increased expression of LepRb in the amygdala. We believe that the increase in LepRb in the amygdala stimulates the hyperphagic effect of leptin, which results in lower weight, weight variation, and plasma leptin concentration. More research is needed to clarify the mechanism of action of leptin in the context of chronic social stress, including the involvement of the possible binding of leptin to dopaminergic receptors.

## Data availability statement

The original contributions presented in the study are included in the article/**Supplementary Material**. Further inquiries can be directed to the corresponding authors.

## Ethics statement

The animal study was approved by Animal Use Ethics Committee of the Oswaldo Cruz Institute (CEUA/IOC) under license number CEUA/IOC-032/2019-A1. The study was conducted in accordance with the local legislation and institutional requirements.

## Author contributions

RM: Conceptualization, Data curation, Formal analysis, Investigation, Methodology, Validation, Writing – original draft, Writing – review & editing, Visualization. RB: Formal analysis, Investigation, Writing – original draft, Writing – review & editing. VO: Investigation, Writing – original draft. GM: Writing – review & editing, Supervision. SH: Writing – review & editing, Investigation, Methodology, Writing – original draft. AH-P: Conceptualization, Resources, Writing – review & editing. TA-J: Conceptualization, Resources, Writing – review & editing, Funding acquisition. VF: Supervision, Writing – review & editing, Conceptualization, Formal analysis, Investigation, Methodology, Project administration, Resources, Validation, Writing – original draft.
