# Supplementary figures and images for: Influence of leptin and its receptors on individuals under chronic social stress behavior
Source: Front Endocrinol (Lausanne). 2024 Feb 1;15:1281135. doi: 10.3389/fendo.2024.1281135 (PMC10867138; doi:10.3389/fendo.2024.1281135)

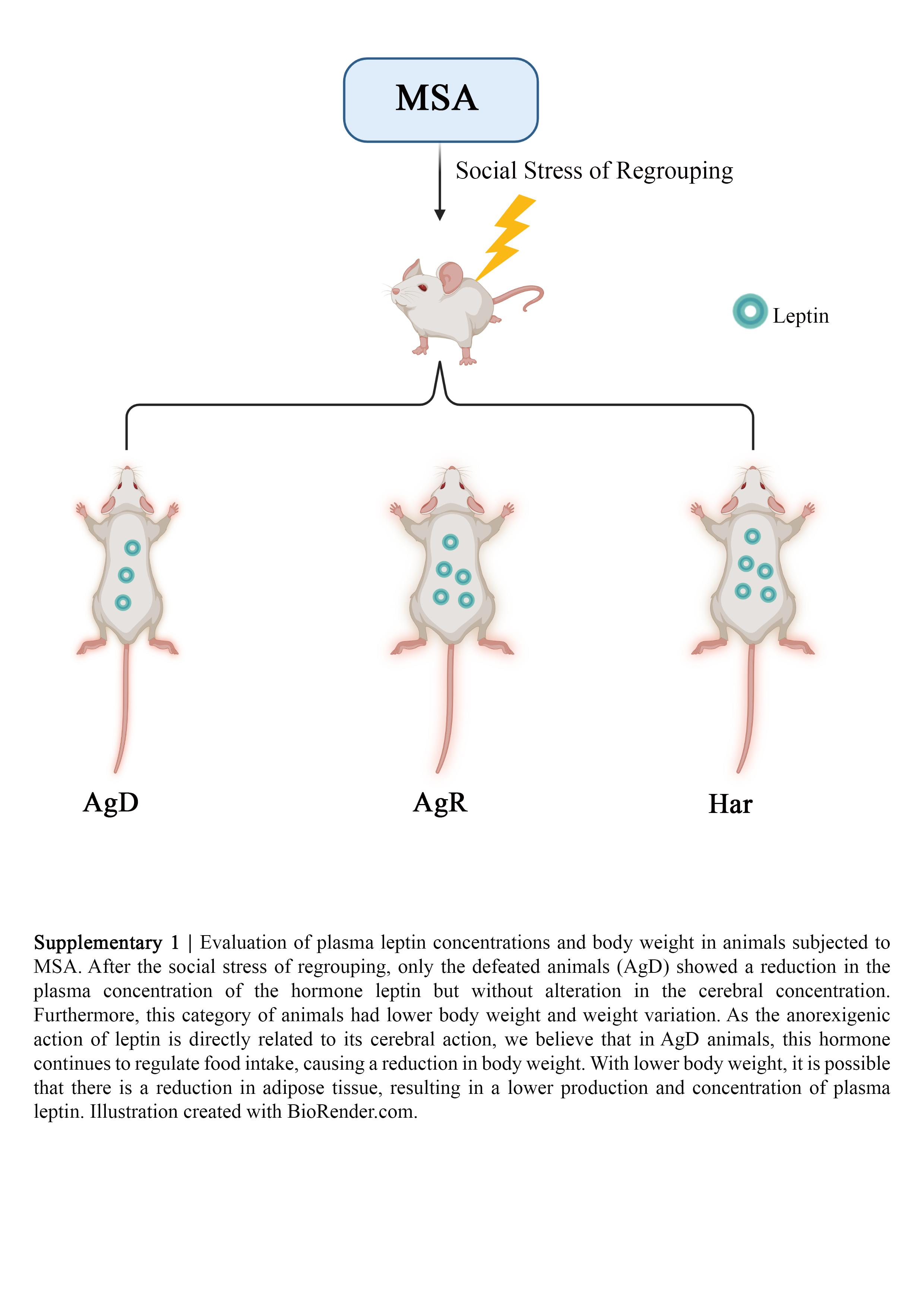

Supplement: Supplementary file 1 [file Image_1.tif]

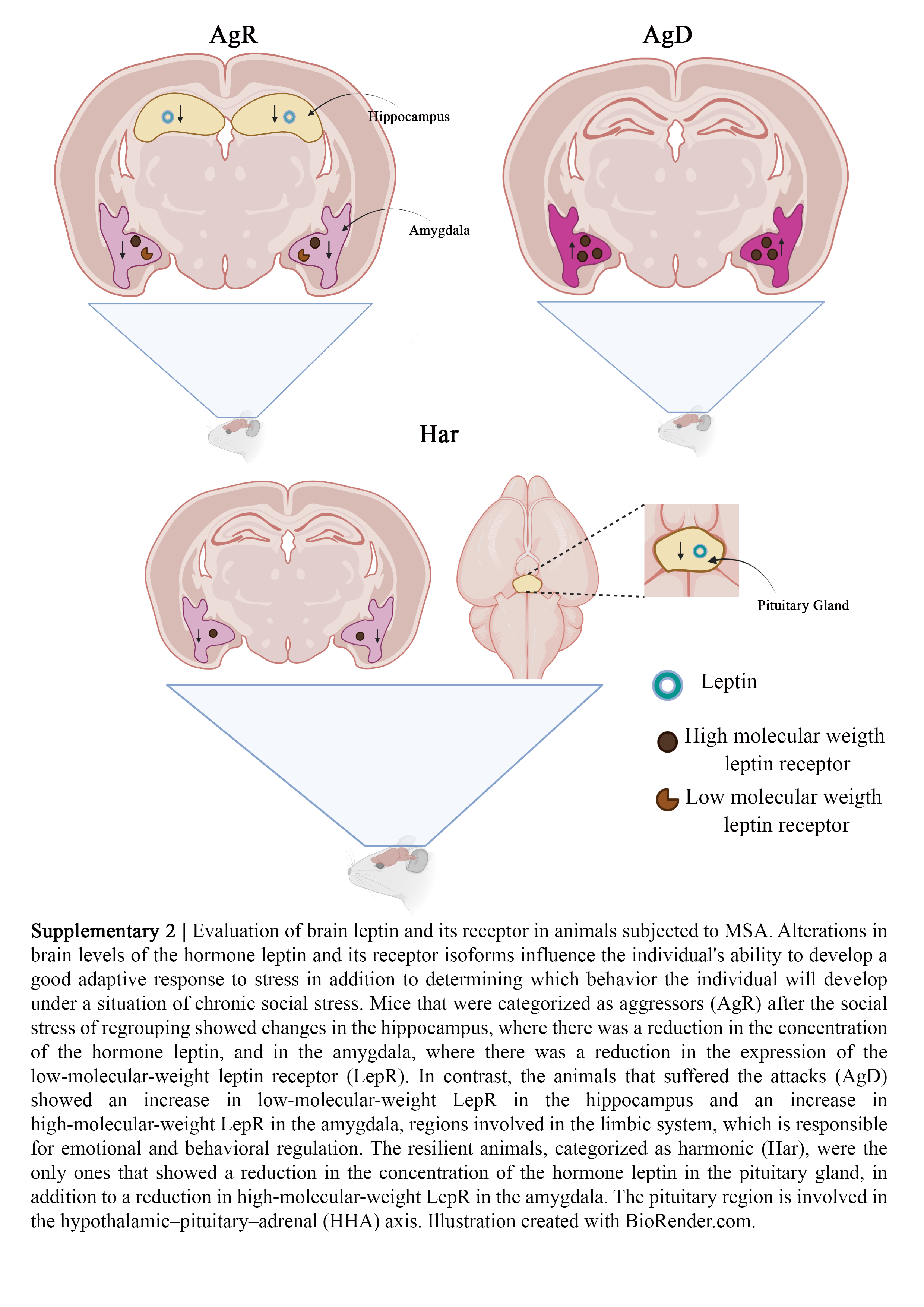

Supplement: Supplementary file 2 [file Image_2.tif]
